# A likelihood-based procedure for obtaining confidence intervals of disease loci with general pedigree data

**DOI:** 10.1186/1753-6561-1-s1-s106

**Published:** 2007-12-18

**Authors:** Shuyan Wan, Shili Lin

**Affiliations:** 1 Department of Statistics, The Ohio State University, 1958 Neil Avenue, 404 Cockins Hall, Columbus, Ohio 43210, USA; 2 Merck Research Laboratories, RY80M-162, 126 East Lincoln Avenue, Rahway, New Jersey 07065, USA

## Abstract

We proposed a confidence interval method for disease gene localization by testing every position on each chromosome of interest for its possibility of being a disease locus and including those not rejected into the interval. Three test statistics were proposed to perform the tests, including one based on LOD and two generalized likelihood ratio tests with or without model averaging (GLRT/MA and GLRT). For the statistic based on LOD, an integrated procedure was proposed with an adaptive and an importance sampling component. We also proposed asymptotic approaches based on GLRT and GLRT/MA as alternatives that are much more efficient computationally but depends on the reliability of the limiting distributions. Besides its efficiency, the asymptotic procedure based on GLRT/MA also takes model uncertainty into consideration. Applications of these methods to the Genetic Analysis Workshop 15 (GAW15) rheumatoid arthritis data from the French population gave results that successfully captured the well recognized susceptibility gene HLA*DRB1 to a less than 6 cM, 99% confidence interval with the two asymptotic approaches.

## Background

With the advances in molecular biology, more and more genome-scan data are available for linkage studies. Even in a preliminary genome scan, there is a need to localize a disease gene to as small a chromosomal region as possible without missing the signal of a true disease locus. The LOD support interval approach tends to undercover disease loci unless the linkage signal is extremely strong, and may be further complicated by the difficulty of choosing an appropriate threshold to account for multiplicity adjustment. Lin et al. [1] proposed a confidence set inference (CSI) approach, wherein a confidence interval of a disease locus can be deduced based on the confidence set of markers that are within a preset distance from the disease locus. This approach also alleviates the problem associated with multiple testing.

In this study, instead of deducing a confidence interval, by efficiently testing every position on the chromosome, we obtained a confidence interval of a disease locus with a couple of strategies based on three different statistics that are applicable to general pedigree data. Investigation of the performance of the new approaches by simulation showed that they worked well even when there were only moderate linkage signals. Because initial genome scan for the rheumatoid arthritis data provided by Genetic Analysis Workshop 15 (GAW15) showed moderate to strong linkage signals on chromosome 6, we applied our methods to the three data sets with microsatellite (MS) markers on that chromosome. We compared our results with the traditional 1-LOD and 3-LOD support intervals.

## Methods

### The hypothesis, test statistics, and confidence set

Suppose there is a chromosomal region of length *C *with at most one disease gene in it. We want to localize the gene, if it exists, by constructing a confidence set of its locus with coverage probability *p *such that the exclusion of the true disease locus on the map from the confidence set is controlled at level *α *= 1 - *p*. Because we can only make a type I error at exactly one of the excluded positions, if there is a disease gene on the map, no multiplicity adjustment is needed [1]. By the duality of confidence set and hypothesis testing, this is equivalent to testing the following hypothesis for every position on the chromosome at level *α*:

*H*_0_:*d *= *d*_0 _vs. *H*_*a*_:*d *≠ *d*_0_,

where *d *is the true but unknown map position of a disease gene on the chromosome, and *d*_0_(*d*_0 _∈ [0, *C*]) is the tested map position.

The LOD score, the conventionally used measure of support for linkage versus absence of linkage, can be utilized as a test statistic here, denoted by *λ*_*d*0_:

*λ*_*d*0 _= log_10_L(*d*_0_)/L(∞) = LOD(*d*_0_).

Two alternative test statistics (GLRT: λ*_d0 _and GLRT/MA: *λ*^MA^_*d*0_) that are generalized likelihood ratio based can also be used:

λ*_d0 _= -2LnL(*d*_0_)/L($\hat{d}$) = 4.6[LOD($\hat{d}$) - LOD(*d*_0_)], and *λ*^MA^_*d*0 _= E_*M*_[λ*_d0_] = 4.6[MALOD($\hat{d}$) - MALOD(*d*_0_)],

where LOD($\hat{d}$) is the maximum LOD score maximized over [0, *C*] as well as when *d *is off the map (*d *= ∞). The model averaging LOD score MALOD is defined as

$$\text{MALOD}(d_{0})=\sum_{i=1}^{S}LOD(d_{0}|M_{i})P(M_{i}),$$

which is an average of LOD scores over a set of *S *disease models (*M*_*i *_values) compatible with the data. More specifically, the set of disease models considered are those that are consistent with the identical-by-descent (IBD) probabilities estimated at the hypothesized trait locus or their perturbations. Such a setup not only accounts for model uncertainty associated with the estimated IBDs but also the uncertainty associated with the estimation of the IBDs. The weights assigned to the models, *P*(*Mi*), are bimodal, with those obtained from the IBD estimates getting a larger weight than those from the perturbations. More details can be found in Wan [2].

A confidence set of the disease locus is then constructed by including all of the positions not rejected. Because the distribution of any of the three test statistics under *H*_0 _cannot be found analytically, we used simulation or asymptotic distribution to approximate this null distribution. For the simulation-based approach, data from multiple markers are simulated simultaneously conditional on the affection status and pedigree structure at each hypothesized disease position *d*_0_. Based on the simulated marker data, the null distribution at that hypothesized position is constructed by a Monte Carlo estimate. The test statistic *λ*_*d*0 _is then compared to the null distribution to determine whether *d*_0 _should be included in the confidence set. It is worth emphasizing that at each hypothesized disease position, all marker data (multipoint) are simulated, regardless of the marker interval in which the hypothesized disease locus lies. Because there are an infinite number of putative disease loci to be tested, a practical strategy is needed to discretize the chromosome so that only a finite number of positions need to be tested without compromising the level of coverage. To further improve the computational efficiency of this simulation-based procedure, an importance sampling (IS) component was also proposed. In the following we describe the integrated procedure and the asymptotic approaches.

### An integrated procedure based on LOD

We begin with a broad search of chromosomal regions to be included in or excluded from the confidence set. This broad search strategy is being referred to as our adaptive component of the integrated procedure. Specifically, the chromosome of interest is divided by the genetic markers, and each interval is considered in turn. For each such chromosomal segment, we divide it into two equal halves. For each half, we test the two end points (L and R) and the mid-point (M), and make inference about whether L-M, and/or M-R should be included in/excluded from the confidence set based on properties of the LOD scores, such as unimodality between two markers [2,3]. If inclusion/exclusion decision cannot be made on an interval (L-M or M-R), it is further divided into two equal halves until either a decision about inclusion/exclusion can be made or the length of the segment is less than a preset threshold.

One could have set the threshold to be sufficiently small so that interpolation based on the two end points of any remaining undecided segment would lead to a coverage probability close to the nominal. However, this would be a computationally inefficient procedure due to the need of constructing a large number of simulated null distributions. Instead, we used a relatively coarse grid (leading to a threshold of 1 cM) in the adaptive step and adopted an importance sampling strategy to further refine the remaining segments (all smaller than the threshold) without any additional simulation. Specifically, suppose *d*_0 _is an interior point of one of such segments with the left end point being *d*_*L*_. We would like to test the hypotheses in Eq. (1) to determine whether *d*_0 _should be included in the confidence set. Let

*X = LOD*(*d*_0_) = *log*_10_(*P*(*G|D *= *d*_0_)/*P*(*G|D *= ∞))

denote the random variable corresponding to the LOD score hypothesizing the disease at position *d*_0_, where *G *is the collection of genotypes of all the individuals at all the marker loci. Then the *c.d.f*. of *X *can be written as:

$$\begin{array}{lll} P(X\leq x) & = & E_{P(G|D=d_{0})}[I(\log_{10}(P(G|D=d_{0})/P(G|D=\infty ))\leq x)]\\ & = & \sum_{G}I(P(G|D=d_{0})\leq 10^{x}P(G|D=\infty ))[P(G|D=d_{0})/P(G|D=d_{L})]P(G|D=d_{L}). \end{array}$$

Thus, the *c.d.f*. of the LOD score at *d*_0 _can be estimated by

$$\hat{P}(X\leq x)=\frac{1}{N}\sum_{i=1}^{N}I(P(G^{i}|D=d_{0})\leq 10^{x}P(G^{i}|D=\infty ))[P(G^{i}|D=d_{0})/P(G^{i}|D=d_{L})],$$

which makes use of the *N *sets of simulated marker data (*G*^*i *^values) with the disease locus hypothesized to be at *d*_*L*_. Note that these simulated marker data are available from the adaptive component step, and thus no additional simulations are needed. The importance sampling weight, *P*(*G*^*i*^|*D *= *d*_0_)/*P*(*G*^*i*^|*D *= *d*_*L*_), can be shown to equal to $10^{LOD(d_{0})-LOD(d_{L})}$ after some algebra, and thus can be easily calculated. We then proceed to test the inclusion/exclusion of *d*_0 _based on this estimated null distribution. Our simulation study [2] indicated accurate estimation with substantial gains in computational efficiency because no additional simulations are needed to estimate distributions at all interior points of a segment after the adaptive step. Additional efficiency can be gained by using the simulated marker data at the right end point as well [2].

### Asymptotic approaches based on GLRT and GLRT/MA

When the sample size is moderate or large and/or when the family structure is not extremely heterogeneous, we can approximate the null distribution of the GLRT by a $\chi_{1}^{2}$ distribution. Thanks to its computational efficiency, one can further take model uncertainty into account by considering the test statistic GLRT/MA, where we approximate its limiting distribution by a weighted sum of independent $\chi_{1}^{2}$ values, with a cautionary note that the actual asymptotic distribution may be more complicated due to the dependency of the component $\chi_{1}^{2}$ values.

## Results

We applied both the integrated procedure and the asymptotic method, with or without model averaging, to the rheumatoid arthritis data from GAW15. Three populations are available with MS marker data, namely the French (FR), North American Rheumatoid Arthritis Consortium (NARAC), and United Kingdom (UK), where the NARAC data consist of general pedigrees and the other two sets are of nuclear families. Prior information showed that there was a well recognized susceptibility gene HLA*DRB1 on chromosome 6. Thus, we focused on chromosome 6 and analyzed those three sets of data individually both at 95% and 99% confidence levels. For each population, we performed the analysis using two disease models inferred from each of the data sets. We also applied the two disease models inferred from the NARAC data to the FR data (segment 1 of Table 1), and reciprocally, we utilized those two inferred from the FR data to the NARAC data (segment 2 of Table 1). For the asymptotic approach with model averaging, the disease models being averaged over included those that are consistent with the estimated IBD probabilities and their perturbations. Details of the disease allele frequencies and their penetrances are in Table 1, which shows the performance at 99% confidence level and that of the 3-LOD intervals.

**Table 1:** 99% confidence intervals (CIs) from various procedures^a^

			GLRT/Asymptotic		
				
Pop.	Models^b ^(*P*_*A*_, *f*_*aa*_, *f*_*Aa*_, *f*_*AA*_)	LOD/Integrated	With MA	Without MA	3-LOD
FR	FR1	11.36*	5.29*	5.71*	
	(0.05, 0.031, 0.045, 0.810)	(42.10, 63.00)	(43.46, 48.75)	(43.45, 49.16)	Null
	FR2	18.85*	11 models^c^	5.70*	
	(0.08, 0.030, 0.033, 0.523)	(40.75, 63.55)		(43.13, 48.83)	Null
	NARAC1	18.28*		14.84*	
	(0.10, 0.032, 0.276, 0.920)	(41.55, 70.87)		(41.41, 62.59)	Null
	NARAC2	19.33*		12.36*	
	(0.15, 0.020, 0.216, 0.695)	(40.75, 63.55)		(41.36, 61.41)	Null

NARAC	NARAC1	12.78	12.50*	13.10*	24.16*
	(0.10, 0.032, 0.276, 0.920)	(35.76, 57.96)	(36.21, 52.56)	(35.87, 52.76)	(34.02, 58.18)
	NARAC2	20.23*	38 models^c^	11.97*	22.82*
	(0.15, 0.020, 0.216, 0.695)	(34.98, 60.62)		(36.39, 52.36)	(34.44, 57.26)
	FR1			5.46	12.88
	(0.05, 0.031, 0.045, 0.810)	Null		(35.20, 40.66)	(28.90, 41.78)
	FR2			5.85	19.04
	(0.08, 0.030, 0.033, 0.523)	Null		(35.01, 40.86)	(29.50, 52.17)

UK	UK1	50.56*	27.65*	27.47*	40.18*
	(0.12, 0.014, 0.093, 0.504)	(34.20, 87.58)	(43.06, 72.51)	(41.60, 69.07)	(38.68, 78.86)
	UK2	19.47	15 models^c^	26.27*	38.54*
	(0.09, 0.025, 0.065, 0.830)	(58.83, 78.58)		(44.59, 74.04)	(40.58, 79.12)

^a^For each model and method, we give the length of the confidence set in cM, with a "*" to signify those that contain the HLA*DRB1 locus. We also provide the convex set of the confidence set below the length. For the GLRT/asymptotic procedure with MA, the number of models accounted for is also provided, below the convex set. The 3-LOD intervals are treated as approximate 99% CI [2].

^b^Models {FR1, FR2}, {NARAC1, NARAC2} and {UK1, UK2} are the models consistent with IBD estimates for the FR, NARAC, and UK data, respectively. *A *is the disease allele and *a *the normal allele.

^c^Models for GLRT/MA are inferred from the IBD estimates at the trait locus and their perturbations. They included the models explained in footnote b for individual analysis.

**Figure 1:**
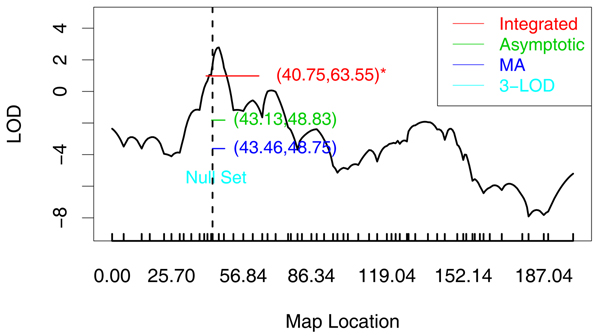
**99% Confidence intervals of French data**. The 99% confidence intervals for rheumatoid arthritis data (French population) analyzed by model FR2. Dashed vertical line is at the HLA*DRB1 locus. *Interval from integrated procedure is a convex set of the original non-contiguous intervals.

Of all three data sets, the 99% integrated procedure and the asymptotic methods successfully captured the HLA*DRB1 locus with at least one of the disease models. All 99% model averaging methods gave intervals containing the HLA*DRB1 locus. Specifically, when there are strong linkage signals as in the NARAC data (maximum LOD scores around 13), at 99% confidence level, our asymptotic methods gave results with shorter length compared to those from the 3-LOD method. Even when there are only moderate signals as in the FR data (maximum LOD around 2.8), at 99% confidence level, the integrated method and the asymptotic methods with or without model averaging all yielded confidence intervals (from 5 to 20 cM) containing the disease locus compared to the null set from the 3-LOD method (Figure 1). Analyses of the UK data also lead to the capturing of the disease locus in all methods, but with lengthier intervals.

Overall, the 95% and 99% asymptotic methods tended to give shorter interval length compared to the corresponding 1-LOD and 3-LOD support intervals when both captured the susceptibility gene. Our methods worked well in most cases, especially when there were moderate linkage signals, such as in the FR data, when the two asymptotic procedures successfully captured the HLA locus to a less than 6-cM 99% confidence region compared to the null set from the 3-LOD support interval approach.

## Discussion

In this paper, we propose three statistics and computational procedures for constructing a confidence set of a disease locus. From our simulation studies [2], the integrated procedure based on the LOD statistic tends to give correct coverage probability and is applicable even when there are minimal linkage signals. The asymptotic method with or without model averaging also works well when there are moderate linkage signals. For example, for the FR data, the model averaging approach localized the putative gene to a 5.29-cM 99% confidence region. When linkage signals are really strong, the integrated procedure tends to give a confidence interval of longer length than the asymptotic methods, when both covered the putative gene, as seen from the application to the NA data. Because the sample size of the NARAC data is large (511 complex families), our asymptotic method with model averaging seemed to work better, giving shorter intervals than its 1-LOD or 3-LOD counterparts. Moreover, the model averaging method is computationally efficient and takes into account of uncertainty in disease model selection. The UK data also have strong linkage signals with a maximum LOD of around 6. However, because the markers around the HLA locus are not very polymorphic and have large inter-marker distances, the integrated method gave wide confidence intervals if the intervals captured the susceptibility gene at all. In this sense, more typed single-nucleotide polymorphisms (SNPs) or MS markers at the region of interest may be helpful in refining the confidence interval. Still, the model averaging method worked relatively well in this case, localizing the susceptibility gene to a 27-cM region. Lastly, due to the computational intensity of the integrated procedure, we presented results only for MS marker data here to compare the performance of our methods with the more traditional *k*-LOD method. With more abundant SNP data now available, the computationally efficient GLRT or GLRT/MA asymptotic procedure would be more applicable.

In conclusion, the GLRT/MA asymptotic procedure is recommended if the sample size is sufficiently large, such as in the GAW data, because it can easily take model uncertainly into account with little additional computational cost. However, when the sample size is relatively small, the asymptotic properties may be questionable, which can lead to shorter confidence intervals with coverage probability lower than its nominal [2].

## Competing interests

The author(s) declare that they have no competing interests.
